# Rapid Eye Movements during REM Sleep Differentiate PSP from Parkinson's Disease

**DOI:** 10.1002/mdc3.14187

**Published:** 2024-08-07

**Authors:** Claudio Togni, Sandra Carpinelli, Philipp O. Valko, Christopher Bockisch, Daniel Waldvogel, Esther Werth, Konrad P. Weber, Yulia Valko

**Affiliations:** ^1^ Department of Neurology University Hospital Zurich Zurich Switzerland; ^2^ Sleep & Health Zurich University Hospital Zurich, University of Zurich Zurich Switzerland; ^3^ Department of Ophthalmology University Hospital Zurich Zurich Switzerland; ^4^ Department of Otorhinolaryngology University Hospital Zurich Zurich Switzerland

**Keywords:** eye movements, rapid eye movement sleep, progressive supranuclear palsy, Parkinson'’ disease, polysomnography

## Abstract

**Background:**

Little is known about the characteristics and occurrence frequencies of rapid eye movements (REMs) during REM sleep in movement disorders.

**Objectives:**

The aim of this study was to detect and characterize REMs during polysomnographically defined REM sleep as recorded by electro‐oculography (EOG) in 12 patients with progressive supranuclear palsy (PSP), 13 patients with Parkinson's disease (PD) and 12 healthy controls.

**Methods:**

Using a modified EOG montage, we developed an algorithm that automatically detects and characterizes REMs during REM sleep based on their presumptive saccadic kinematics.

**Results:**

Compared to PD and healthy controls, REM densities and REM peak velocities were significantly reduced in PSP. These effects were most pronounced in vertical REMs.

**Conclusion:**

Ocular motor dysfunction, one of the cardinal features of PSP, seems to be equally at play during REM sleep and wakefulness. For future studies, we provide a novel tool for the unbiased analysis of REMs during REM sleep in movement disorders.

Ocular motor abnormalities present a unique window to neural networks primarily located within the brainstem. This region is particularly affected in neurodegenerative parkinsonian syndromes, such as Parkinson's disease (PD) and progressive supranuclear palsy (PSP).^1^, ^2^

Neurodegeneration in PSP involving the brainstem tegmentum leads to marked ocular motor dysfunction, the most profound of which is slowing and hypometria of vertical saccades, which eventually progress to vertical gaze palsy.^3^, ^4^, ^5^ Hence, ocular motor abnormalities play a crucial role in distinguishing PSP from PD on clinical grounds.

Similarly, the neural network responsible for generating rapid eye movement (REM) sleep – including its key characteristics, REM sleep atonia and REMs—is located within the brainstem, too.^6^ Indeed, the pathophysiological underpinning of REM sleep behavior disorder (RBD), a frequent and early feature of PD and other alpha‐synucleinopathies, is thought to lie in the neurodegeneration of these caudal brainstem structures.^7^ The frequency of RBD in PD, up to 60%, is much higher than in PSP.^8^ Accordingly, diagnosing RBD by polysomnography (PSG) provides an additional tool for differentiating PD and other alpha‐synucleinopathies from PSP and other tauopathies.

While electro‐oculography (EOG) is an integral part of PSG, providing crucial information on sleep stages, little attention has been paid to the characteristics and occurrence frequencies of eye movements during sleep. Here, we hypothesized that the frequency and characteristics of REMs differ between PD and PSP and developed a simple and computationally inexpensive algorithm to detect and characterize REMs based on their presumptive saccadic kinematics using standard polysomnography recordings with a modified EOG electrode montage.

## Methods

### Patients

A total of 25 patients, of whom 13 had PD and 12 had PSP, and 12 healthy controls (HC) were prospectively enrolled in this study. Diagnosis of PSP was considered probable (n = 11) or possible (n = 1) according to the diagnostic criteria for clinical diagnosis of PSP set forth by The Movement Disorder Society.^9^ Most PSP patients presented clinically evident ocular motor abnormalities typical of PSP at the time of PSG, including slowed vertical saccades (n = 4) or vertical supranuclear gaze palsy (n = 6). Detailed participants' characteristics can be found in Supplementary Table S1.

One PD patient and two PSP patients exhibited no REM sleep during the PSG night and were excluded from subsequent analyses.

### Polysomnography and Electro‐Oculography

To differentiate horizontal and vertical eye movements, a modified electrode montage including two electrodes lateral to and at the level of each outer canthus for horizontal eye movements, and one electrode above the right eye and one electrode below the right eye for vertical eye movements, was used (Supplementary Figs. S1A, S2A and S3A). All electrodes were referenced to an ipsilateral mastoid electrode, respectively. Other than that, a conventional 60‐channel polysomnography system was used, and manual sleep scoring was performed as recommended by the American Academy of Sleep Medicine (AASM).^10^

### Detection of Rapid Eye Movements

After preprocessing, referenced EOG signals were segmented into phases of equal direction, and each of these phases was considered to contain a potential REM. Based on presumed saccadic kinematics of REMs, each of these phases was then serially passed through eight selection criteria. Only those fulfilling all selection criteria were accepted as REMs and classified as horizontal or vertical REMs. Whenever a horizontal and a vertical REM overlapped, they were considered as one oblique REM. Oblique REMs were only included in the evaluation of REM density, but not in the evaluation of amplitude, duration and peak velocity. To avoid noise amplification, REMs were then modeled by fitting a generalized logistic function onto them. Modeled amplitudes, durations and peak velocities were then used for group‐wise comparisons.

**Figure 1:**
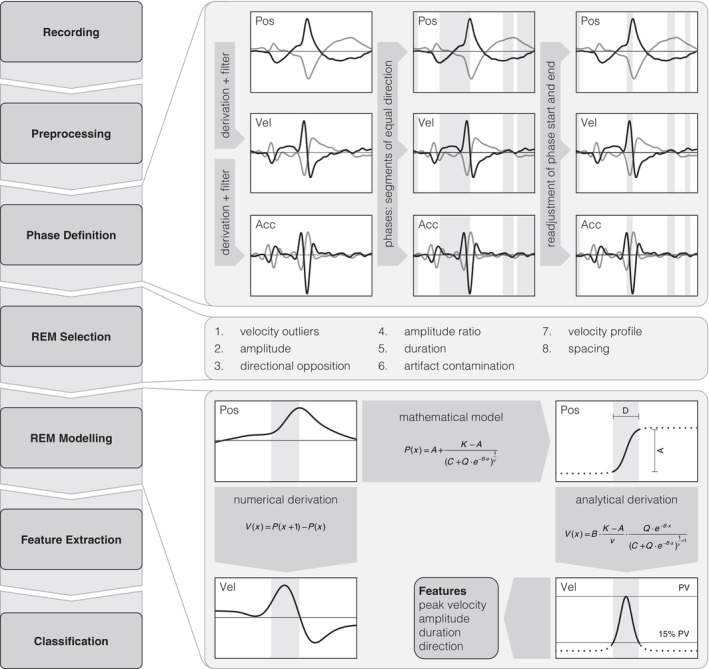
Graphical overview of the rapid eye movement (REM) detection algorithm. The left side shows the general workflow of the algorithm. Raw electro‐oculography (EOG) traces are referenced and filtered, resulting in a position signal (Pos), and corresponding velocity (Vel) and acceleration (Acc) signals are acquired by numerical derivation and subsequent filtering. Phases are defined as segments of equal direction according to the sign of the velocity signal, and phase boundaries are readjusted to the timepoints of maximum and minimum acceleration (upper right). These phases are then passed through rapid eye movement (REM) selection criteria (see supplementary text for details), and only those phases fulfilling all selection criteria are considered to represent true REMs (middle right). The example shows an epoch of 5 s duration, containing three phases representing potential horizontal REMs (gray bars). Only the first of these fulfills all of the REM selection criteria and is therefore considered to represent a true REM. The filtered position signal of such a REM is then mathematically modeled using a generalized logistic function and the corresponding velocity signal is obtained by analytical derivation rather than numerical derivation. REM features, such as peak velocity (PV), amplitude (A), duration (D) and direction are extracted from the modeled REM (lower right). The variables of the generalized logistic function are: A, lower asymptote; K, upper asymptote; C = 1; Q, variable related to the inflection point; B, growth (velocity) rate; v, timepoint of maximum growth (velocity).

Figure 1 provides a graphical overview of the REM detection algorithm. Its detailed description and examples of REMs as recorded by the modified EOG montage and detected by the algorithm for a 30s epoch of REM sleep of a PSP patient, a PD patient, and a healthy control can be found in the Supplementary Material File S1, and Supplementary Figs. S1, S2 and S3, respectively.

### Statistics

The Kruskal‐Wallis test and Fisher's least significant difference test as a post‐hoc test were used to compare mean ranks of REM features between groups. Statistical significance was accepted at *P* < 0.05. All statistical analyses were performed and illustrated graphically using custom‐built code based on MATLAB version R2021a (The Mathworks, Inc., Natick, MA, USA).

## Results

### Rapid Eye Movement Density

**Figure 2:**
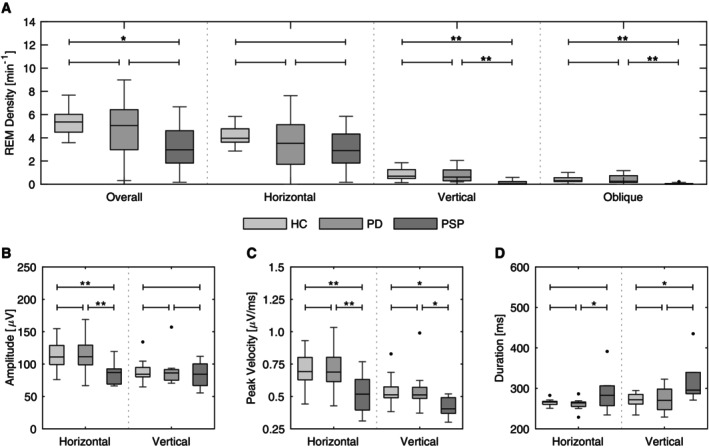
Statistical comparison of REM characteristics between patients with Parkinson's disease (PD), progressive supranuclear palsy (PSP) and healthy controls (HC) as traditional box plots for horizontal and vertical REMs. (**A**) Overall, horizontal, vertical and oblique REM densities. (**B**) Mean amplitudes given in μV. (**C**) Mean peak velocities given in μV/ms. (**D**) Mean durations given in ms. Statistically significant results are marked by *P < 0.05; and **P < 0.01.

REM density was calculated as the number of REMs per minute of analyzed REM sleep. In one PD patient, no horizontal REMs were detected, and in two PSP patients, no vertical REMs were detected. Vertical and oblique REM densities were significantly reduced in PSP patients when compared to healthy controls (*P* = 0.0007 and *P* = 0.0005, respectively) and to PD patients (*P* = 0.0009 and *P* = 0.0020, respectively). This was also reflected by reduced overall REM densities in PSP patients compared to healthy controls (*P* = 0.0147). On the other hand, the groups did not differ in horizontal REM density. In contrast to PSP, there was no difference in overall, horizontal, vertical, or oblique REM density of PD patients compared to those of healthy controls (Fig. 2A).

### Rapid Eye Movement Amplitude, Peak Velocity, and Duration

Mean amplitudes of horizontal REMs were significantly smaller in PSP patients than in healthy controls and PD patients (*P* = 0.0033 and *P* = 0.0031, respectively), while no such effect was seen for vertical REMs (Fig. 2B). However, mean peak velocities were significantly reduced in PSP patients when compared to healthy controls and PD patients for both horizontal (*P* = 0.0044 and *P* = 0.0054, respectively) and vertical REMs (*P* = 0.0409 and *P* = 0.0447, respectively) (Fig. 2C). Mean durations of horizontal and vertical REMs of PSP patients were significantly longer than those of PD patients (*P* = 0.0353) and healthy controls (*P* = 0.0443), respectively (Fig. 2D). On the other hand, neither horizontal nor vertical REMs of PD patients and healthy controls showed any significant differences in amplitudes, peak velocities or durations (Fig. 2B–D).

## Discussion

EOG remains the only method to date to study eye movements during sleep. Meanwhile, only little attention has been paid to the characteristics and occurrence frequencies of REMs during REM sleep despite the former being a defining feature of the latter. Here, we developed a simple and computationally inexpensive algorithm for automated detection and characterization of REMs during REM sleep that rapidly provides results concerning REM density and REM characteristics. The algorithm was subsequently applied to patients with PD, PSP, and healthy controls.

REM density was significantly reduced in PSP when compared to PD and healthy controls. This was in large part caused by a reduction of vertical REMs (Fig. 2A). Similarly, mean peak velocities of REMs were significantly lower in PSP than in PD and healthy controls, too (Fig. 2C). These findings suggest that the pathophysiological mechanisms underlying supranuclear gaze palsy preferentially affecting vertical eye movements in PSP are equally at play during wakefulness and sleep. Interestingly, in our study, PD patients did not show significantly different REM densities or REM characteristics compared to healthy controls.

Several methodological drawbacks with regard to EOG in general, as well as to the detection algorithm, must be considered when interpreting the results of this study. The signal intensity obtained by EOG recording is individual and direct comparison of interindividual REM amplitudes is difficult. On a group basis, as performed in this study, however, comparing patient groups may still show valid results. Furthermore, the REM selection criteria—the most significant of them being the amplitude of a phase—may introduce a relevant selection bias towards larger REMs. In addition, the measures of REM amplitude, duration, and peak velocity, as provided by the algorithm, may only be regarded as approximations to the true kinematic features of REMs. Finally, while EOG and PSG represent elegant research tools, their use in current clinical practice for the workup of movement disorders is restricted to specialized, resource‐rich centers.

In summary, the algorithm developed for this study provides a novel tool for fast and unsupervised analysis of REMs during REM sleep and hence, avoids subjectivity and interrater bias in detecting REMs. It may be used for investigating the brainstem‐centered interplay of ocular motor function and sleep in a multitude of disease states and may contribute to the elucidation of critical aspects of REM sleep physiology, e.g., the distribution of phasic and tonic REM sleep in health and disease, a field that has recently gained increasing interest.^11^ With regard to the differentiation between PSP and PD, we provide evidence for the usefulness of our algorithm at advanced, clinically unambiguous disease stages. Sufficiently powered prospective studies with eye movement measurements during sleep and wakefulness will be needed to determine its diagnostic yield at early disease stages.

## Author Roles

(1) Research project: A. Conception, B. Organization, C. Execution; (2) REM detection algorithm: A. Development, B. Review and critique; (3) Statistical analysis: A. Design, B. Execution, C. Review and critique; (4) Manuscript: A. Writing of first draft, B. Review and critique.

C.T.: 1C, 2A, 3A, 3B, 4A,B

S.C.: 1B, 1C, 4B

P.O.V.: 1A, 1B, 1C, 4B

C.B.: 2B, 3C, 4B

D.W.: 1A, 1B, 1C, 4B

E.W.: 1B, 1C, 4B

K.P.W.: 1A, 1B, 2B, 3C, 4B

Y.V.: 1A,1B, 1C, 2B, 3C, 4B

## Disclosures

**Ethical Compliance Statement:** The study protocol was approved by the Ethics Committee of the Canton of Zurich (KEK‐ZH‐Nr. 2017–01323), and the study was conducted in accordance with the Declaration of Helsinki. Prior to study enrollment, all participants gave written informed consent. The authors confirm that they have read the Journal's position on issues involved in ethical publication and affirm that this work is consistent with those guidelines.

**Funding Sources and Conflict of Interest:** The study was funded by a grant from the Olga Mayenfisch Foundation. The authors declare that there are no conflicts of interest relevant to this work.

**Funding Disclosures for the Previous 12 Months:** YV was supported by the Betty and David Koetser Foundation for Brain Research. KW was supported by the Swiss National Science Foundation [320030_166346] and the Uniscientia Stiftung, Vaduz, Liechtenstein. EW was supported by the Swiss National Science Foundation [320030_160075].

## Supporting information


**Figure S1.** Example of a 30s epoch of REM sleep in a patient with progressive supranuclear palsy (PSP). (**A**) Modified electro‐oculography montage. (**B**) Hypnogram showing the distribution of wakefulness (W), REM sleep (R) and non‐REM sleep 1, 2 and 3 (N1, N2 and N3), respectively, over the course of the night. (**C**) Polysomnography showing electro‐oculography (EOG), electroencephalogram (EEG) and electromyogram (EMG) of the chin and the four limbs. (**D**) EOG traces after preprocessing. Arrowheads point to REMs as they may be scored by visual inspection. The gray bars mark REMs as detected by the REM detection algorithm. Note that there is a potential small horizontal REM at approximately 24 s that is not detected by the algorithm due to its low amplitude.

**Figure S2.** Example of a 30s epoch of REM sleep in a patient with Parkinson's disease (PD). (**A**) Modified electro‐oculography montage. (**B**) Hypnogram showing the distribution of wakefulness (W), REM sleep (R) and non‐REM sleep 1, 2 and 3 (N1, N2 and N3), respectively, over the course of the night. (**C**) Polysomnography showing electro‐oculography (EOG), electroencephalogram (EEG) and electromyogram (EMG) of the chin and the four limbs. (**D**) EOG traces after preprocessing. Arrowheads point to REMs as they may be scored by visual inspection. The gray bars mark REMs as detected by the REM detection algorithm. In the vertical traces (lower part), note that the algorithm cannot reliably differentiate between the REM and the following signal suppression due to alternate current (AC)‐coupling which may contain a REM itself (as in to‐and‐fro saccades).

**Figure S3.** Example of a 30s epoch of REM sleep in a healthy control (HC). (**A**) Modified electro‐oculography montage. (**B**) Hypnogram showing the distribution of wakefulness (W), REM sleep (R) and non‐REM sleep 1, 2 and 3 (N1, N2 and N3), respectively, over the course of the night. (**C**) Polysomnography showing electro‐oculography (EOG), electroencephalogram (EEG) and electromyogram (EMG) of the chin and the four limbs. (**D**) EOG traces after preprocessing. Arrowheads point to REMs as they may be scored by visual inspection. The gray bars mark REMs as detected by the REM detection algorithm.

**TABLE S1.** Participants’ characteristics. Statistically significant differences are marked by *P < 0.05 and **P < 0.01. HC, healthy controls; PD, Parkinson's disease; PSP, progressive supranuclear palsy; y, years; MDS‐UPDRS III, Unified Parkinson's Disease Rating Scale by the Movement Disorder Society, Part III; LEDD, levodopa equivalent daily dose; mg, milligrams; REM, rapid eye movement sleep; RSWA, REM sleep without atonia; RBD, REM sleep behavior disorder.^1^ REM sleep behavior disorder according to the International Classification of Sleep Disorders (Sateia, 2014).^2^ Diagnostic level of certainty according to The Movement Disorder Society Criteria for the clinical diagnosis of progressive supranuclear palsy Höglinger et al.^9^

**File S1.** Detailed description of the algorithm for rapid eye movement detection.

## Data Availability

The data that support the findings of this study are available from the corresponding author upon reasonable request.
