# Supplementary figures and images for: Rapid Eye Movements during REM Sleep Differentiate PSP from Parkinson's Disease
Source: Mov Disord Clin Pract. 2024 Aug 7;11(10):1281–5. doi: 10.1002/mdc3.14187 (PMC11489613; doi:10.1002/mdc3.14187)

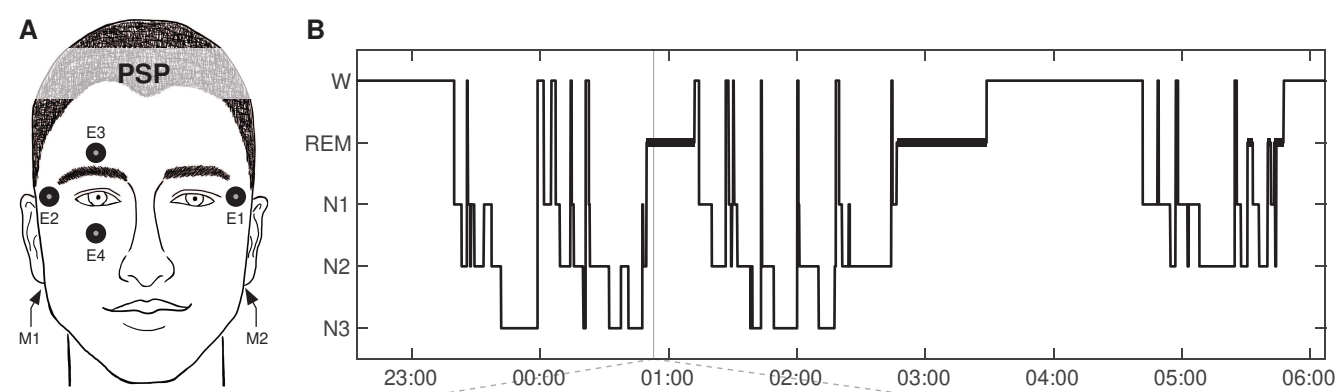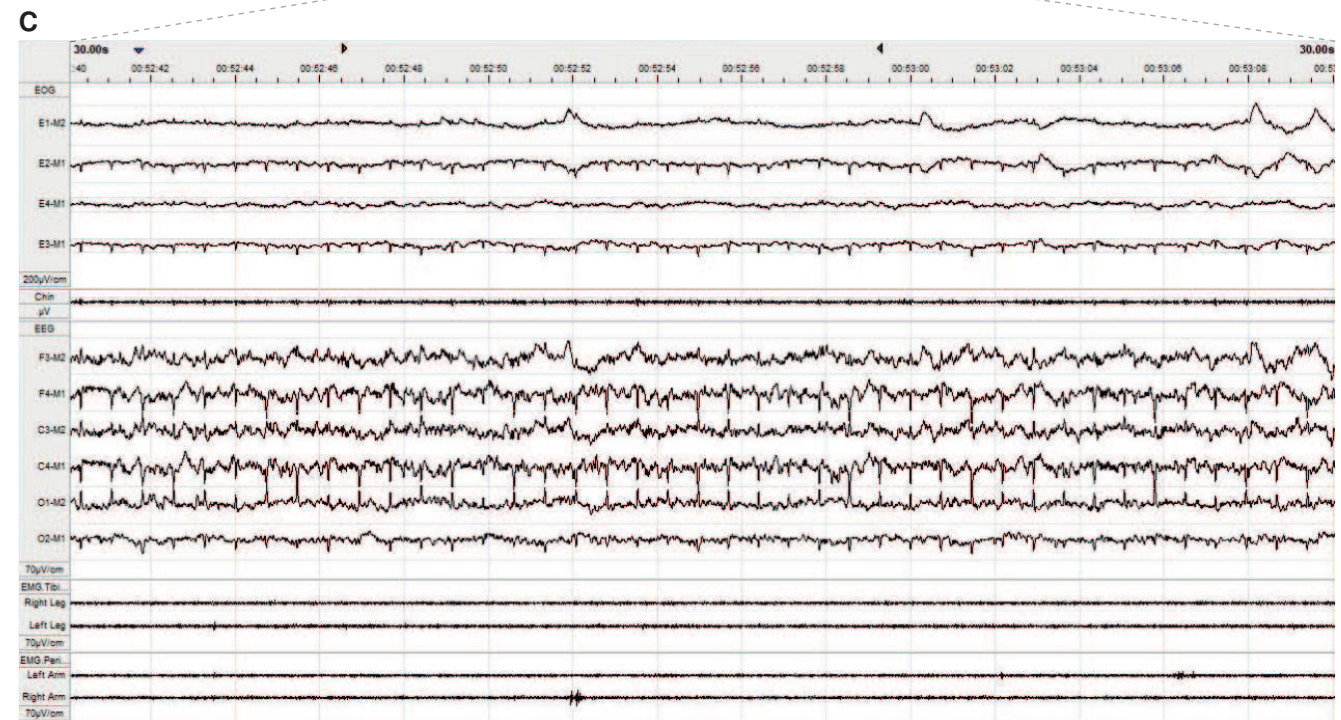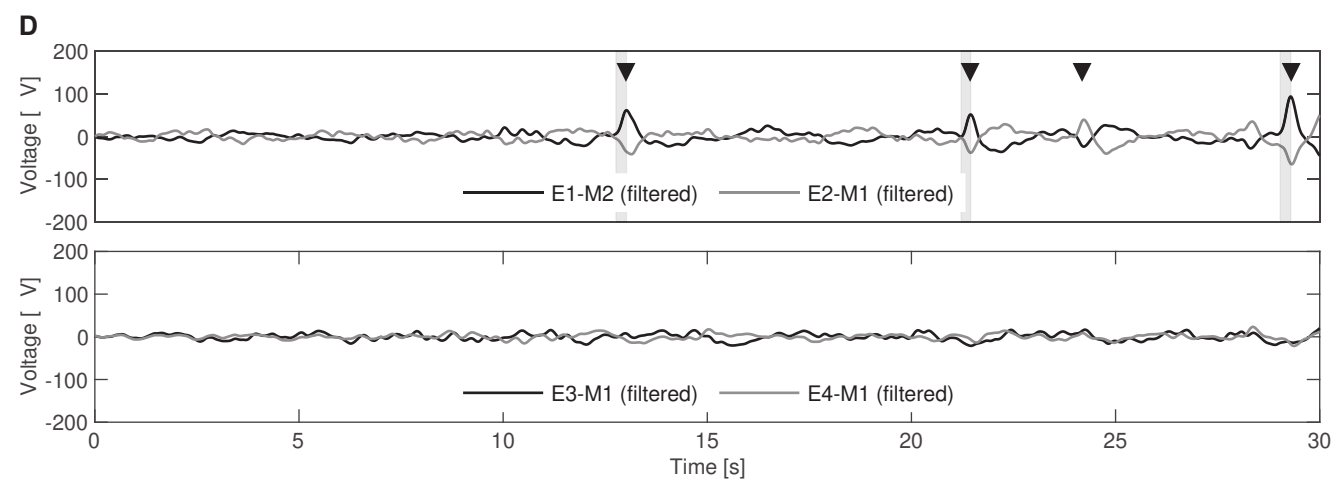

Supplement: Supplementary file 1 — Figure S1. Example of a 30s epoch of REM sleep in a patient with progressive supranuclear palsy (PSP). (A) Modified electro‐oculography montage. (B) Hypnogram showing the distribution of wakefulness (W), REM sleep (R) and non‐REM sleep 1, 2 and 3 (N1, N2 and N3), respectively, over the course of the night. (C) Polysomnography showing electro‐oculography (EOG), electroencephalogram (EEG) and electromyogram (EMG) of the chin and the four limbs. (D) EOG traces after preprocessing. Arrowheads point to REMs as they may be scored by visual inspection. The gray bars mark REMs as detected by the REM detection algorithm. Note that there is a potential small horizontal REM at approximately 24 s that is not detected by the algorithm due to its low amplitude. [file MDC3-11-1281-s005.pdf]

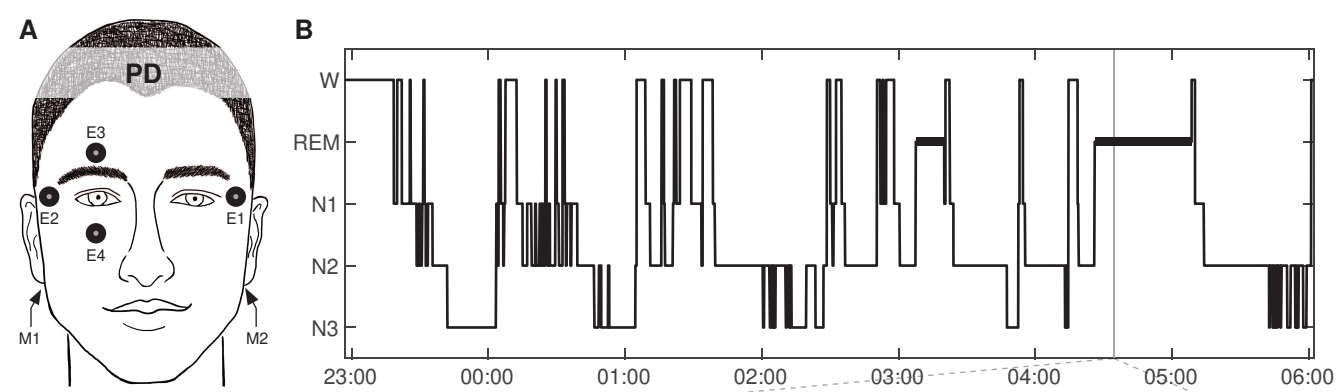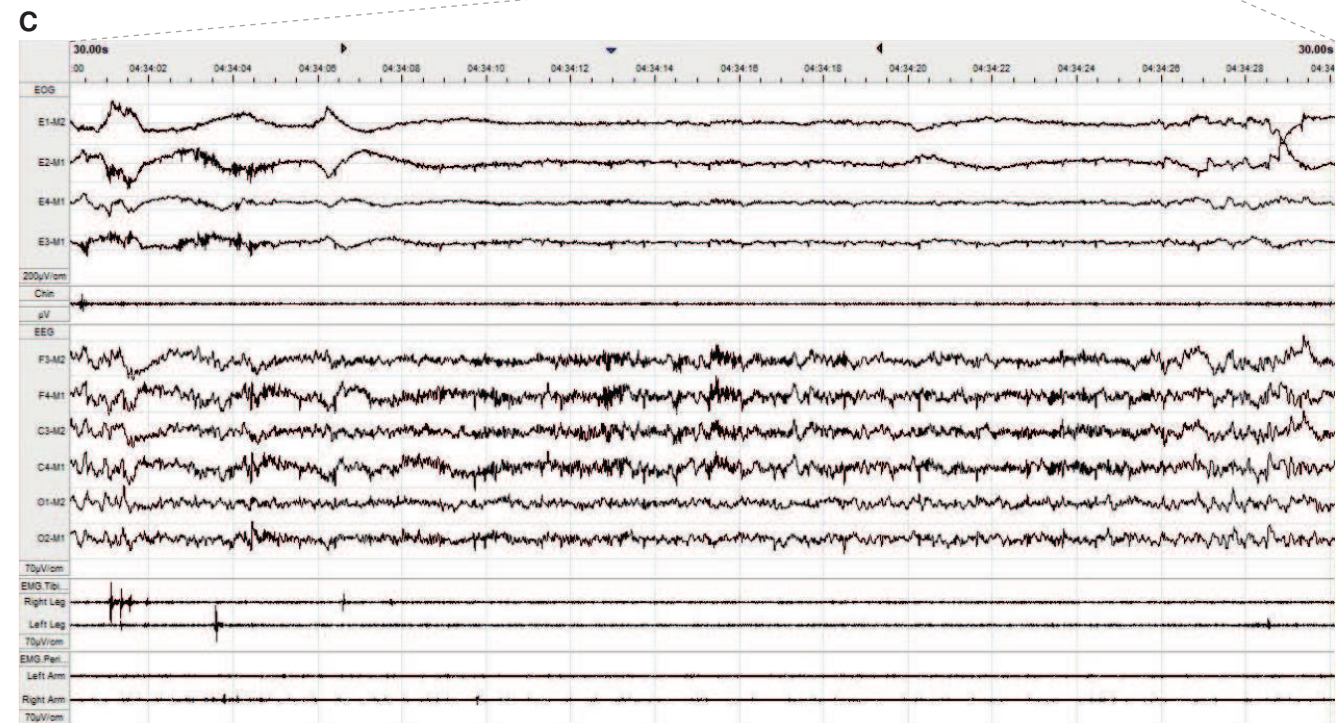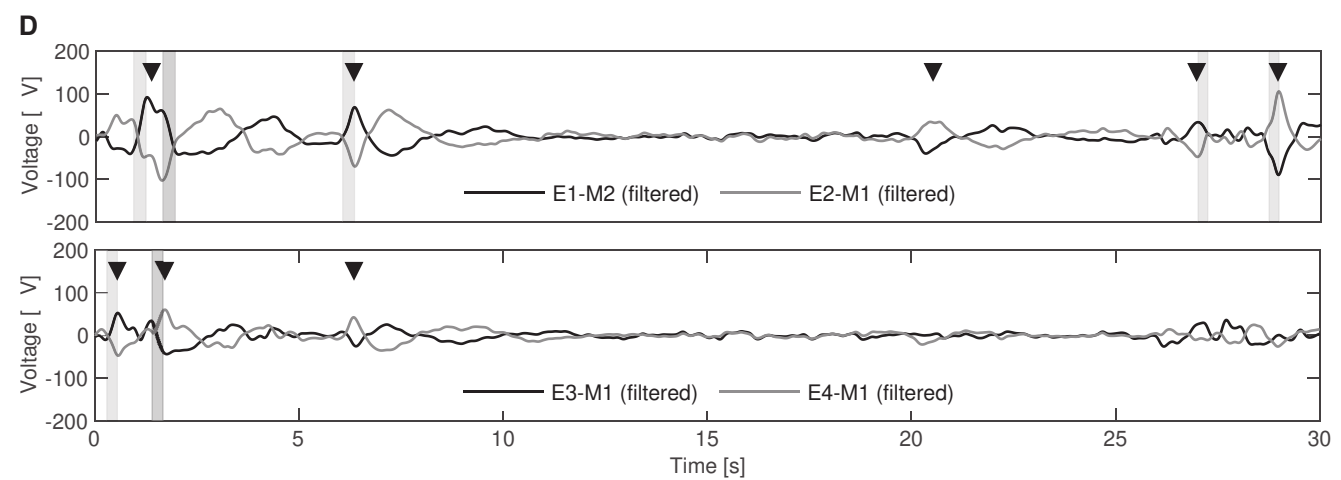

Supplement: Supplementary file 2 — Figure S2. Example of a 30s epoch of REM sleep in a patient with Parkinson's disease (PD). (A) Modified electro‐oculography montage. (B) Hypnogram showing the distribution of wakefulness (W), REM sleep (R) and non‐REM sleep 1, 2 and 3 (N1, N2 and N3), respectively, over the course of the night. (C) Polysomnography showing electro‐oculography (EOG), electroencephalogram (EEG) and electromyogram (EMG) of the chin and the four limbs. (D) EOG traces after preprocessing. Arrowheads point to REMs as they may be scored by visual inspection. The gray bars mark REMs as detected by the REM detection algorithm. In the vertical traces (lower part), note that the algorithm cannot reliably differentiate between the REM and the following signal suppression due to alternate current (AC)‐coupling which may contain a REM itself (as in to‐and‐fro saccades). [file MDC3-11-1281-s002.pdf]

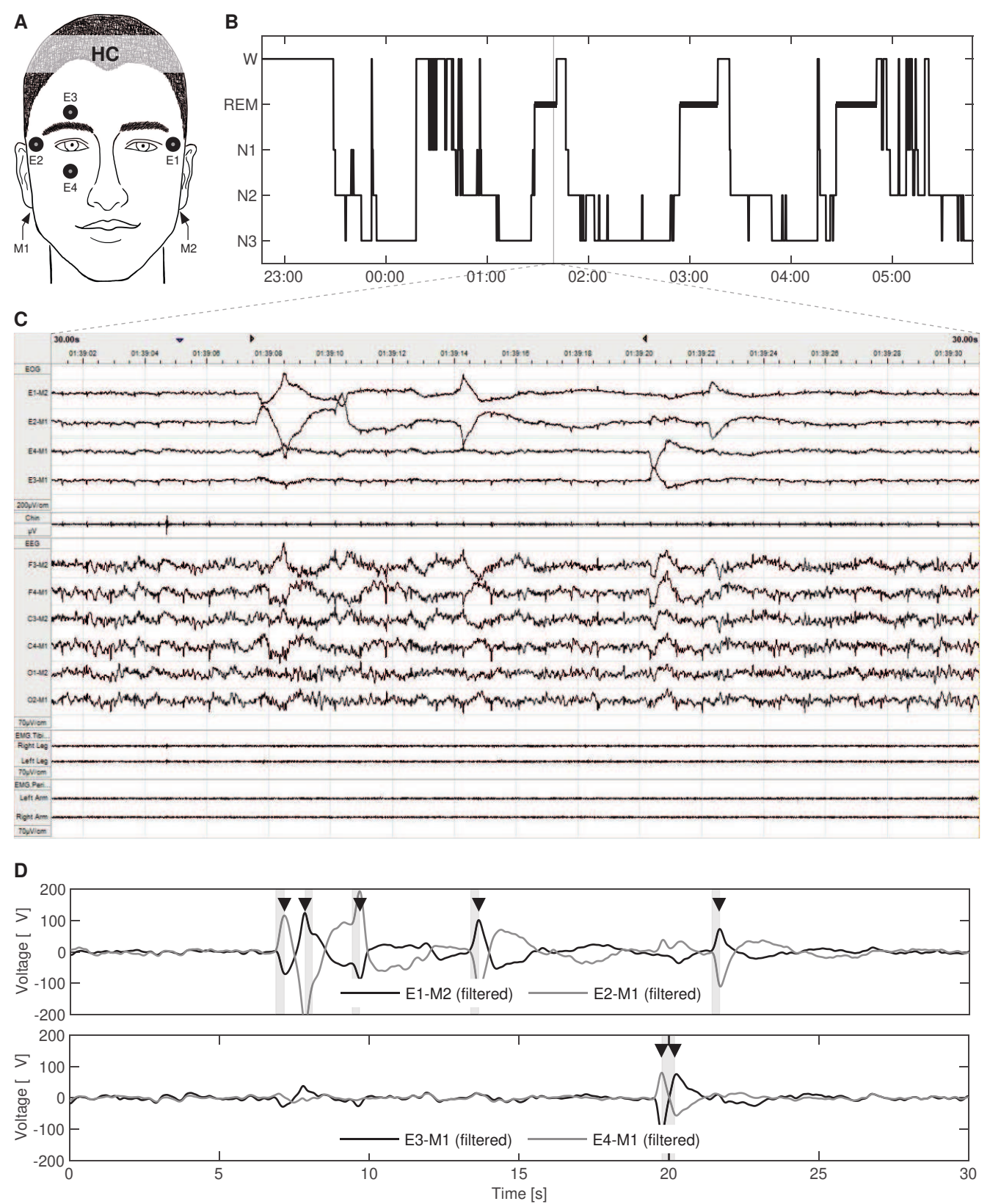

Supplement: Supplementary file 3 — Figure S3. Example of a 30s epoch of REM sleep in a healthy control (HC). (A) Modified electro‐oculography montage. (B) Hypnogram showing the distribution of wakefulness (W), REM sleep (R) and non‐REM sleep 1, 2 and 3 (N1, N2 and N3), respectively, over the course of the night. (C) Polysomnography showing electro‐oculography (EOG), electroencephalogram (EEG) and electromyogram (EMG) of the chin and the four limbs. (D) EOG traces after preprocessing. Arrowheads point to REMs as they may be scored by visual inspection. The gray bars mark REMs as detected by the REM detection algorithm. [file MDC3-11-1281-s001.pdf]
