# Supplementary material for: Rapid Eye Movements during REM Sleep Differentiate PSP from Parkinson's Disease
Source: Mov Disord Clin Pract. 2024 Aug 7;11(10):1281–5. doi: 10.1002/mdc3.14187 (PMC11489613; doi:10.1002/mdc3.14187)
Supplement: Supplementary file 4 — TABLE S1. Participants’ characteristics. Statistically significant differences are marked by *P < 0.05 and **P < 0.01. HC, healthy controls; PD, Parkinson's disease; PSP, progressive supranuclear palsy; y, years; MDS‐UPDRS III, Unified Parkinson's Disease Rating Scale by the Movement Disorder Society, Part III; LEDD, levodopa equivalent daily dose; mg, milligrams; REM, rapid eye movement sleep; RSWA, REM sleep without atonia; RBD, REM sleep behavior disorder.1 REM sleep behavior disorder according to the International Classification of Sleep Disorders (Sateia, 2014).2 Diagnostic level of certainty according to The Movement Disorder Society Criteria for the clinical diagnosis of progressive supranuclear palsy Höglinger et al. 9 [file MDC3-11-1281-s003.docx]

**Rapid Eye Movements During REM Sleep Differentiate PSP from Parkinson's Disease**

**– Supplementary Material –**

Claudio Togni^1^, MD, Sandra Carpinelli^1^, MD, Philipp O. Valko^1,2^, MD, Christopher Bockisch^1,3,4^, PhD, Daniel Waldvogel^1^, MD, Esther Werth^1,2^, PhD, Konrad P. Weber^1,3^, MD, Yulia Valko^1^, MD

|  | HC (n = 12) | PD (n = 13) | PSP (n = 12) | p |
| --- | --- | --- | --- | --- |
| *Demographics* |  |  |  |  |
| Age [y], mean (min - max) | 62 (50 - 73) | 64 (51 - 83) | 70 (59 - 82) | 0.0415 * |
| Gender, male/female | 8/4 | 8/5 | 4/8 |  |
| *Disease Severity* |  |  |  |  |
| Disease duration [y], mean (min - max) | n.a. | 7 (1 - 21) | 4 (1 - 10) | 0.1146 |
| MDS-UPDRS III, mean (min - max) | n.a. | 23 (7 - 41) | 33 (11 - 53) | 0.1106 |
| LEDD [mg], mean (min - max) | n.a. | 687 (0 - 1620) | 340 (0 - 1500) | 0.0372 * |
| *REM sleep behavior disorder* |  |  |  |  |
| Isolated RSWA, n (%) | 0 (0) | 5 (38) | 3 (25) |  |
| RBD ^1^, n (%) | 0 (0) | 4 (31) | 1 (8) |  |
| Disease subtype |  |  |  |  |
| PD, tremor-dominant type, n (%) | n.a. | 4 (30.8) | n.a. | n.a. |
| PD, akinetic-rigid type, n (%) | n.a. | 5 (38.5) | n.a. | n.a. |
| PD, mixed type, n (%) | n.a. | 4 (30.8) | n.a. | n.a. |
| PSP, Richardson‘s syndrome, n (%) | n.a. | n.a. | 8 (66.7) | n.a. |
| PSP, predominant parkinsonism, n (%) | n.a. | n.a. | 3 (25.0) | n.a. |
| PSP, not specified, n (%) | n.a. | n.a. | 1 (8.3) | n.a. |
| Ocular motor features |  |  |  |  |
| Vertical supranuclear gaze palsy, n (%) | n.a. | n.a. | 6 (54.5) | n.a. |
| Slowing of vertical saccades, n (%) | n.a. | n.a. | 4 (36.3) | n.a. |
| Other, n (%) | n.a. | n.a. | 1 (0.9) | n.a. |
| Diagnostic level of certainty ^2^ |  |  |  |  |
| Probable PSP, n (%) | n.a. | n.a. | 11 (91.7) | n.a. |
| Possible PSP, n (%) | n.a. | n.a. | 1 (8.3) | n.a. |

Supplementary Table 1: Participants' characteristics. Statistically significant differences are marked by * (p < 0.05) and ** (p < 0.01). HC, healthy controls; PD, Parkinson’s disease; PSP, progressive supranuclear palsy; y, years; MDS-UPDRS III, Unified Parkinson's Disease Rating Scale by the Movement Disorder Society, Part III; LEDD, levodopa equivalent daily dose; mg, milligrams; REM, rapid eye movement sleep; RSWA, REM sleep without atonia; RBD, REM sleep behavior disorder. ^1^ REM sleep behavior disorder according to the International Classification of Sleep Disorders (Sateia, 2014). ^2^ Diagnostic level of certainty according to The Movement Disorder Society Criteria for the clinical diagnosis of progressive supranuclear palsy (Höglinger et al., 2017).
