# Supplementary material for: Rapid Eye Movements during REM Sleep Differentiate PSP from Parkinson's Disease
Source: Mov Disord Clin Pract. 2024 Aug 7;11(10):1281–5. doi: 10.1002/mdc3.14187 (PMC11489613; doi:10.1002/mdc3.14187)
Supplement: Supplementary file 5 — File S1. Detailed description of the algorithm for rapid eye movement detection. [file MDC3-11-1281-s004.docx]

**Rapid Eye Movements During REM Sleep Differentiate PSP from Parkinson's Disease**

**– Supplementary Material –**

Claudio Togni^1^, MD, Sandra Carpinelli^1^, MD, Philipp O. Valko^1,2^, MD, Christopher Bockisch^1,3,4^, PhD, Daniel Waldvogel^1^, MD, Esther Werth^1,2^, PhD, Konrad P. Weber^1,3^, MD, Yulia Valko^1^, MD

**Detection of Rapid Eye Movements**

*Preprocessing*

EOG traces were recorded with a sampling frequency of 200Hz. The raw traces were loaded into MATLAB R2021a (The MathWorks, Inc., Natick, Massachusetts, USA), and the following steps were performed in MATLAB using custom-built code based on built-in functions. Only REM sleep periods with a duration of at least 180s were considered, and the first and last epochs of 30s duration were truncated to avoid analysis of transitions between REM sleep and non-REM sleep or wakefulness.

The referenced EOG traces, i.e. E1-M2, E2-M1, E3-M1, and E4-M1, corresponding to position signals, were filtered using a second-order zero-phase Butterworth bandpass filter with cut-off frequencies at 0.1Hz and 3.0Hz. Velocity signals, i.e. V(E1-M2), V(E2-M1), V(E3-M1), and V(E4-M1), and acceleration signals, i.e. A(E1-M2), A(E2-M1), A(E3-M1) and A(E4-M1) were obtained by numerical derivation of the filtered position signal and the filtered velocity signal, respectively, and were each filtered again using the same filtering method.

*Phase Definition*

Position signals were segmented into phases of equal direction based on the sign of the corresponding velocity signals. Phase boundaries, i.e. start and end of a phase, were then readjusted to the time points of maximum acceleration and maximum deceleration, respectively, based on the corresponding acceleration signals. Any of these phases were considered to contain a potential REM.

*REM Selection*

Each of these phases was serially passed through eight selection criteria, and only those phases that fulfilled all criteria were considered to represent actual REMs. These selection criteria are largely based on the assumption that REMs exhibit saccadic kinematics and are in line with the AASM's definition of REMs as conjugate, irregular, sharply peaked eye movements with an initial deflection usually lasting less than 0.5s.^9^

1. The phase contains a velocity outlier. Outliers in the corresponding velocity signal were identified using a local-scaled median absolute deviation (MAD)-approach with a moving window of 30s or 6000 samples duration.
2. The phase has an absolute amplitude of at least 50µV.
3. The direction of the phase is opposite to the direction of the opposing trace, i.e. E1-M2 for E2-M1 and vice versa for horizontal EOG and E3-M1 for E4-M1 and vice versa for vertical EOG. For horizontal eye movements, this guarantees that there is an abducting eye movement in one eye and an adducting eye movement in the contralateral eye. However, as only the right eye was subjected to vertical EOG recording, this is only a criterion of plausibility for vertical eye movements, as a change in the retinocorneal potential in the vertical plane due to a vertical eye movement is expected to result in opposing potential differences above and below the eye.
4. The ratio of the phase amplitude and the amplitude of the opposing trace, i.e. E1-M2 for E2-M1 and vice versa for horizontal EOG and E3-M1 for E4-M1 and vice versa for vertical EOG, lies between 0.33 and 3. This guarantees that abducting and adducting amplitudes are similar, i.e. conjugate for horizontal eye movements, while it is, again, only a criterion of plausibility for vertical eye movements.
5. The phase has a duration of 0.5s or less.
6. The phase is not contaminated by high-frequency artifacts, such as movement or muscle artifacts. Only phases containing no spikes bigger than 40µV in a second-order zero-phase high-pass Butterworth filtered copy of the position signal are considered (cut-off frequency: 50Hz).
7. The phase has a parabolic velocity profile. A second-order polynomial curve is fitted onto the velocity signal, and only phases with a goodness-of-fit R^2^ of at least 0.6 are considered.
8. There is an interval of at least 0.5s between two adjacent REMs. If the intersaccadic interval is shorter than 0.5s, the two adjacent saccades are rated according to the critical characteristics amplitude (2), amplitude ratio (4), duration (5), and velocity profile (7) (one point for each: amplitude > 125µV, duration < 0.2s, goodness-of-fit R^2^ > 0.8, amplitude ratio > 0.8 and < 1.2). The phase with the lower rating is omitted. This step is necessary because alternate current (AC)-coupled EOG recording produces signal suppression segments immediately after REMs that closely resemble REMs themselves. This is exemplified in Supplementary Figures 2D and 3D.
9. *REM Modelling.* Despite filtering, EOG data is noisy and numerical derivation for corresponding velocity, and acceleration signals will inherently amplify noise. Hence, the measurement of dynamic features of REMs, in particular peak velocity, based on EOG, is imprecise. Furthermore, due to the low sampling frequency, REM peak velocities and REM amplitudes may be underestimated, while REM durations may be over- or underestimated. To address these imprecisions, the position signals of REMs were modeled mathematically by fitting a generalized logistic function onto them. Analytical derivation of the fitted model yields a velocity signal robust to noise. The corresponding formulas for the position and velocity signals can be found in Figure 1.

*Feature Extraction*

For each of these REMs, the features duration, amplitude, peak velocity, and direction of the modeled REM are stored.

*Classification*

REMs are classified based on their primary direction as horizontal or vertical REMs, respectively. Whenever a horizontal and a vertical REM overlap, they are considered as one oblique REM. Oblique REMs are included in the evaluation of temporal REM features (i.e. REM density), but are excluded in the evaluation of dynamic features (i.e. amplitude or peak velocity) to avoid additional imprecision.

The plausibility of the results given by the algorithm, i.e. the detected REMs, was extensively checked by visual inspection of the corresponding polysomnography and EOG traces.
